# Experimental data on electrical properties of epoxy/carbon composites used as structural capacitance

**DOI:** 10.1016/j.dib.2019.104867

**Published:** 2019-11-22

**Authors:** Eric Yurman, Kedar Kirane

**Affiliations:** Mechanical Engineering, Stony Brook University, United States

**Keywords:** Woven composites, Carbon fibres, Structural capacitance, Multifunctional composites, Electromechanical composites

## Abstract

This data article reports experimentally measured electrical properties of a plain-woven epoxy/carbon fabric composite used as structural capacitance. The composite laminate was fabricated via the hand lay-up technique with a polyethylene (PET) film sandwiched between the layers. The composite layers acted as electrodes while the PET film acted as the dielectric separator. The electrical properties of this composite laminate capacitor were measured by connecting it to an automatic LCR meter via copper connectors. The properties measured included the series and parallel resistance, series and parallel capacitance and the capacitance density. The data allows assessment of the electrical performance of this composite when fabricated via the hand lay-up method. The data can be used for comparison with similar or other composites, employing the same or more sophisticated fabrication techniques, and for assessing the composite's multifunctional capabilities.

**Table:** Specifications Table

Subject	Engineering, Material science
Specific subject area	Woven composites, carbon fibres, structural capacitance, multifunctional composites, electromechanical composites
Type of data	Table Electrical properties
How data were acquired	Philips PM6303A LCR meter
Data format	Raw
Parameters for data collection	Specimens were made of 3K plain-woven carbon fabric with epoxy resin matrix. The dielectric separator was a polyethylene (PET) film. The specimens were prepared using the hand layup method. The data collected included series and parallel resistance, series and parallel capacitance and the capacitance density of the composite specimens.
Description of data collection	The data were measured by connecting the specimens to the LCR meter via copper connectors. The measurements were conducted at 1 kHz frequency.
Data source location	Institution: Stony Brook University City/Town/Region: Stony Brook, NY, USA Country: USA Latitude: 40.913479 and Longitude −73.125507
Data accessibility	With the article

**Table:** 

**Value of the Data** • The data allow assessing the potential of epoxy/carbon woven composites for use as structural capacitance, especially when fabricated by the hand layup technique • Engineers and researchers investigating carbon fibre composites as a multifunctional material to carry out mechanical and electrical functions can benefit from this data • This article outlines the procedure for fabricating good quality composite laminate specimens by hand layup technique when including polyethylene (PET) film inserts • The data provided here are potentially the lower bound of the electrical properties, and can be used for comparison with similar or other multifunctional composites fabricated via hand layup or other more sophisticated techniques

## 1 Data

The data presented here include the electrical properties of the epoxy/carbon fabric plain woven composite laminate specimens, intended to be used as structural capacitance and fabricated by the hand layup technique. Hand lay-up is the simplest and most economical composites moulding method and also the crudest. As such the electrical properties of specimens thus prepared can be considered to be a lower bound, with improvements expected with increasing sophistication of the fabrication process. The specimens consist of 4 layers with one PET film sandwiched between the second and third layer. Each pair of the carbon fabric layers acts as an electrode while the PET film acts as the dielectric separator. The electrical properties considered are series and parallel resistance, series and parallel capacitance and capacitance density.

## 2 Experimental design, materials, and methods

The composites were fabricated by using 3K plain woven carbon fabric purchased from Fiber Glast Inc (3k indicates 3000 filaments per fibre). The resin used was the system 2000 epoxy resin and the curing agent was the 2120 two-hour epoxy cure, both purchased from Fiber Glast Inc as well. A Polyethylene terephthalate (PET) film was used as the dielectric separator and was 0.05 mm or 50 μm thick (trade name: DuPont Mylar A).

**Fig. 1:**
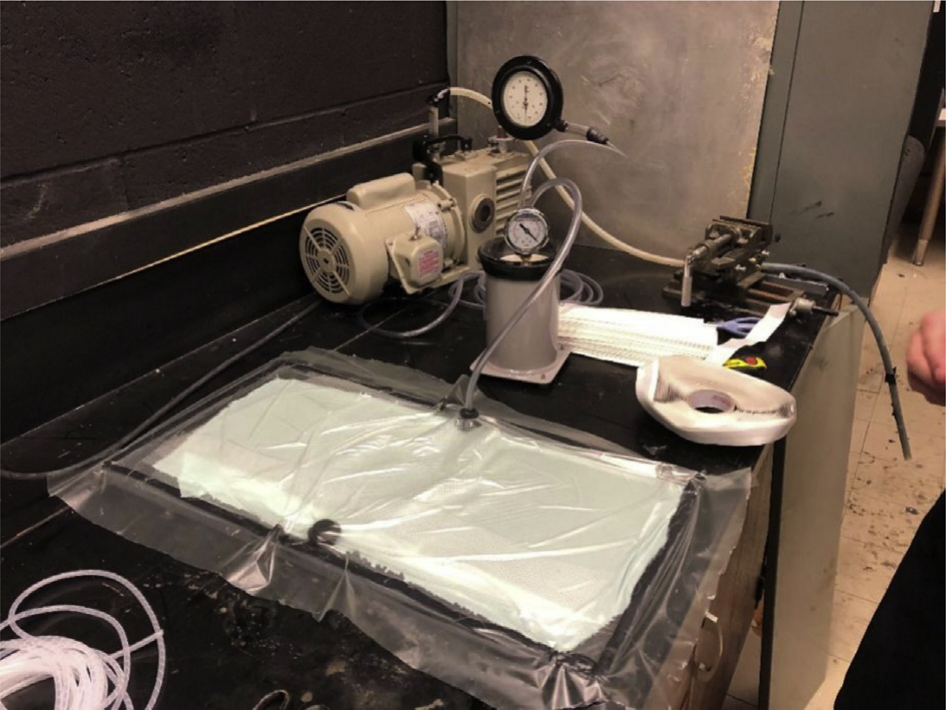
Setup used for fabricating the composite laminate specimens.

The epoxy resin was mixed with the hardener in a ratio of 100:27 by weight as specified by the supplier. Then the carbon fabric layers were placed by hand one by one on a flat panel lined with the peel ply. The carbon fabric layers were approximately 200 mm × 450 mm in size. The resin/hardener mixture was applied onto each layer with the aid of a brush. Between the second and third layer, a PET film was added. The PET film was placed so that it was fully contained within the carbon fabric layers. The stack of these layers was then vacuum bagged, sealed and connected to a General Electric 1/3 HP vacuum pump through airtight tubes. The vacuum pressure was maintained between 25- and 30-inches Hg and the system was left to cure for 48 hours. The setup is shown in Fig. 1.

**Fig. 2:**
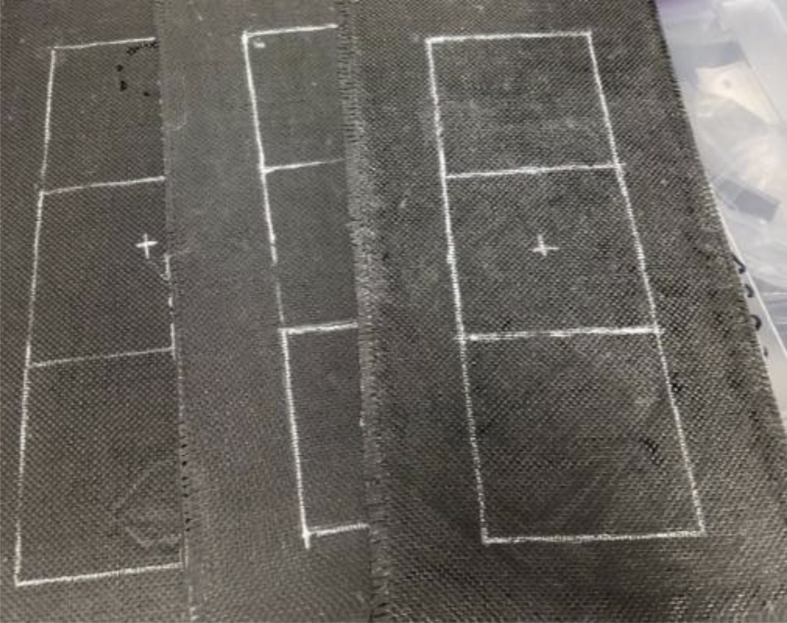
Specimens just after curing with the to be cut specimen shapes marked with chalk.

The cured composite laminates with a total thickness of 1.3 mm were removed from the vacuum bagging. Square shaped specimens of dimension 110 × 110 mm were cut out from these cured laminates for electrical testing. The cutting operation was conducted by using an industrial shearing machine. The to be cut shapes of the specimen are as shown on the cured laminates in Fig. 2.

**Fig. 3:**
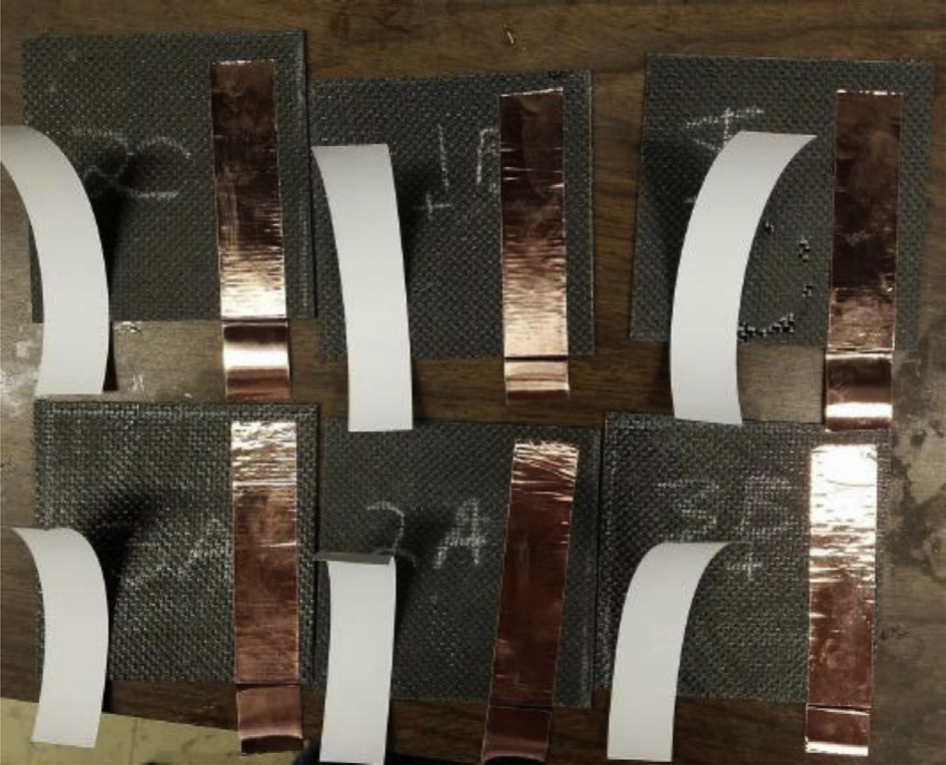
Specimens cut and taped, ready for electrical measurements.

Out of the total nine specimens prepared, three specimens exhibited issues with lack of adhesion between the carbon fabric and the PET layer especially during the cutting operation. These specimens were discarded, and characterization was performed with only the remaining six good quality specimens. For the electrical measurements two strips of conductive copper tape were adhered to opposite sides of the specimens, spaced so that any interference with each other was eliminated, as shown in Fig. 3.

**Fig. 4:**
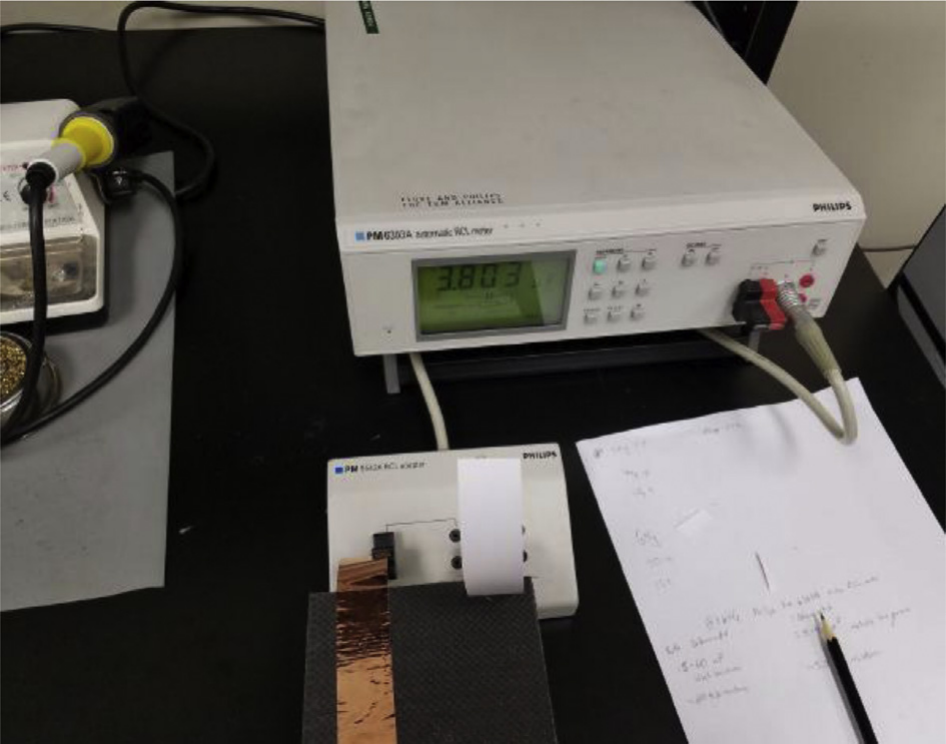
Experimental setup showing one of the specimens connected to the LCR meter.

The specimens were then connected by means of copper connectors to a Philips PM6303A automatic LCR meter which measured their capacitance and resistance values. The measurements were taken by reading the specimen's capacitance and resistance at 1 kHz, readily provided by the LCR meter. Both series and parallel measurements were conducted. The LCR meter also provided the capacitance density values. A sample measurement being taken, is shown in Fig. 4.

The measured data is shown in Table 1 for all six specimens. The average values and the standard deviation are shown as well.

**Table 1:** Table of measured electrical properties of all six specimens.

Specimen	Cp	Rp	Cs	Rs	Capacitance density
	pF	(MOhm)	F	Ohm	nF/m2
1	250	10	225	8	20.7
2	200	33	190	25	16.5
3	388	23	387	7.5	32.1
4	338	27	338	13	27.9
5	370	28	372	6.5	30.6
6	425	3	417	51	35.1
Average	328.50	20.67	321.50	18.50	27.15
Standard deviation	86.42	11.64	92.56	17.34	7.14

Although there is some variation in the electrical properties, most specimens are seen to fall within the same range of values for all electrical properties. This consistency affirms that the fabrication procedure was repeatable. It is noted that the capacitance density values seen here are ∼10 times lower than previously reported for similar material systems [1,2] but fabricated using pre-preg layers.
